# Molecular karyotypes of loquat (*Eriobotrya japonica*) aneuploids can be detected by using SSR markers combined with quantitative PCR irrespective of heterozygosity

**DOI:** 10.1186/s13007-020-00568-7

**Published:** 2020-02-24

**Authors:** Guo Wen, Jiangbo Dang, Zhongyi Xie, Jinying Wang, Pengfei Jiang, Qigao Guo, Guolu Liang

**Affiliations:** College of Horticulture and Landscape Architecture; Academy of Agricultural Sciences; Key Laboratory of Horticulture Science for Southern Mountain Regions of Ministry of Education; State Cultivation Base of Crop Stress Biology for Southern Mountainous Land, Southwest University, Beibei, Chongqing, China

**Keywords:** Loquat, Aneuploidy, SSR markers, qPCR, Molecular karyotype

## Abstract

**Background:**

Aneuploidy, a condition caused by an imbalance between the relative dosages of chromosomes, generally produces a novel phenotype specific to the molecular karyotype. Few techniques are currently available for detecting the molecular karyotypes of aneuploids in plants.

**Results:**

Based on this imbalance in chromosome dosage, a new approach (referred to as ‘SSR-qPCR’) combining simple sequence repeat (SSR) markers and quantitative real-time PCR (qPCR) has been developed and utilized to detect some common aneuploids irrespective of heterozygosity. We screened 17 specific SSR markers covering all loquat linkage groups and redesigned 6 pairs of primers for SSR markers that can detect loquat chromosome aneuploidies. The SSR-qPCR detection results obtained for hybrid progeny and open-pollination progeny of triploid loquat showed diagnostic accuracies of 88.9% and 62.5%, respectively, compared with the chromosome preparation results.

**Conclusion:**

SSR-qPCR can detect loquat aneuploids and be used to construct the entire molecular karyotypes of aneuploid individuals. Therefore, this method offers a novel alternative for the detection of chromosome aneuploidies.

## Background

Aneuploidy can result in partial genome duplication, which involves the imbalance between the relative dosages of chromosomes [1]. Aneuploidy is usually lethal in plants [2–4], and the surviving individuals frequently exhibit novel phenotypic traits specific to the molecular karyotypes. Each of the 12 *Datura* trisomies results in a different phenotype, depending on which type of chromosome has trisomy [2]. Henry et al. [1] demonstrated that certain traits are strongly associated with the dosages of specific chromosome types and that chromosomal effects can be additive in *Arabidopsis thaliana*. Some aneuploids are useful for crop production. Guava, pear and loquat aneuploids have been studied in relation to rootstock selection for dwarfing [5–9]. Moreover, aneuploidy in plants is associated with some unique and desirable traits, such as multiple petals, male sterility, tolerance of cold or drought or resistance to disease [10–12]. Therefore, research on aneuploidy is of great significance for crop production. The identification of aneuploids and the construction of their complete molecular karyotypes are fundamental and crucial for such research.

Many techniques have been widely used for the detection of chromosome copy number changes in aneuploid individuals. The unquestionable and unique merit of classic cytogenetics and fluorescence in situ hybridization (FISH) is their abilities to produce complete karyotypes and reveal balanced major rearrangements; however, these techniques are time consuming and labour intensive [13]. Flow cytometry does not accurately detect aneuploidy, but simple sequence repeat (SSR) and single nucleotide polymorphism (SNP) markers are advantageous for the reliable identification of aneuploidy [14]. Many recently developed DNA-based methods are faster, less expensive and achieve a higher resolution compared with the previously available methods. Quantitative fluorescent polymerase chain reaction (QF-PCR) is widely applied in humans for the prenatal diagnosis of trisomy of chromosomes 21, 18, 13, X, and Y [15, 16]. SNP arrays have been used as high-throughput genotyping tools for polyploid wheat, potato, sugarcane, and strawberry crops [17]. Quantitative real-time polymerase chain reaction (qPCR) has also been used for detection of the trisomy of chromosomes 21 (Down syndrome) and 11 [18]. qPCR, due to its specificity, sensitivity and replicability, is widely used for gene expression analysis and is one of the most reliable tools for the detection of nucleic acid sequence copy numbers [13, 19]. However, the application of qPCR for the detection of aneuploid plant molecular karyotypes has rarely been reported. Previously, aneuploid and polyploid *A. thaliana* have been detected by measurement of the relative allelic ratios of the heterozygous genotype [3]. The details of polyploidization have been described in *Malus*, with particular emphasis on aneuploidy, via detection of the types of heterozygous genotypes and the corresponding frequencies of occurrence [12]. Both of these methods are limited to heterozygous genotypes; in particular, the detection of the occurrence frequencies of heterozygous genotypes using statistical methods requires large numbers of codominant SSR markers. Similarly, high-resolution melting (HRM) and multiplex ligation-dependent probe amplification (MLPA) are also limited to heterozygous genotypes. SSR markers are randomly distributed throughout the plant genome and are reproducible, codominant, and transferable [20]. Microsatellite markers have been widely used in genetic and paternal studies [21, 22], but they are rarely used in molecular karyotypic studies. Codominant markers can be amplified from allelic loci simultaneously by qPCR. When allelic loci are homozygous, the ratios between markers from different loci are constant between euploids. If there are appropriate SSR markers in each linkage group (LG) used for qPCR, accurate detection of aneuploidy based on the whole-chromosome dosage can be achieved through the use of qPCR combined with SSR markers to distinguish aneuploidy irrespective of heterozygosity. Notably, with the publication of the genome sequences of major crops, more SSR markers have become available.

Loquat (*Eriobotrya japonica* (Thunb.) Lindl.) originated in Southwest China, and among the 20 species belonging to the genus *Eriobotrya*, only loquat is used for commercial fruit production [23, 24]. Many triploid lines that can be derived from unreduced gametes have been selected from progeny of diploid germplasms [25], and some aneuploid plants can be derived from triploids [1, 4]. These aneuploid loquat plants are useful for research on the effects of chromosome dosage on the genomic expression and phenotypes of plants. At present, the whole molecular karyotype of aneuploid loquat is helpful for genomics-based research.

In this study, we found, for the first time, that a combination of SSR markers and qPCR (SSR-qPCR) can be used to construct the entire molecular karyotypes of aneuploid individuals of *E. japonica*. This approach does not require large numbers of SSR markers or multiple types of fluorescent markers. In addition, this DNA-based qPCR method does not require additional reference genes, and SSR markers in 17 LGs can be selected as cross-references. The approach is also not restricted to heterozygous genotypes. Moreover, SSR-qPCR allows the construction of molecular karyotypes without whole-genome sequence data, and then validated in the offspring populations of triploid lines, and more than 88% of the offspring of triploids were found to be aneuploid. These data will be critical for the development of molecular tools and strategies for loquat breeding programmes.

## Methods

### Plant material

**Fig. 1:**
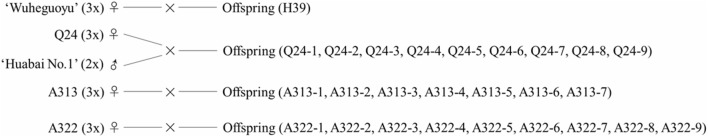
Illustration of the known and unknown pedigrees of some accessions. ♀indicates the female parent, ♂ indicates the male parent, and × indicates the crossing or seeding progeny

Twenty-three loquat lines with different ploidies were used as the materials in this study (Table 1): 2 Sichuan Province cultivars (‘Dawuxing’ and ‘Longquan No. 1’), 2 Zhejiang Province cultivars (‘Ruantiaobaisha’ and ‘Ninghaibai’), 1 Fujian Province cultivar (‘Changbai No. 1’) and 18 excellent strains selected at Southwest University (A313, A322, B350, B352, B353, B356, B431, B456, B460, B432, ‘Wuheguoyu’, H424, H39, 77-1, K474, ‘Huabai No. 1’, Q24 and ‘Huayuwuhe No. 1’). All of these lines were obtained from the polyploid loquat germplasm resource nursery at Southwest University, Chongqing, China. In addition, to produce aneuploid strains, we performed hybridization of Q24 (3x) × ‘Huabai No. 1’ (2x), and we selected some hybrids and elucidated their genetic relationships (Fig. 1). Moreover, we found that two triploid loquat strains (A313 and A322) produced seeds by open pollination, and we selected their progeny as additional research materials (Fig. 1).

**Table 1:** Twenty-three known loquat strains used in this study

Code	Strain	Ploidy	Origin
1	‘Dawuxing’	2n = 2x= 34	Sichuan, China
2	A313	2n = 3x = 51	Selected from ‘Dawuxing’
3	A322	2n = 3x = 51	Selected from ‘Dawuxing’
4	‘Longquan No. 1’	2n = 2x = 34	Sichuan, China
5	B350	2n = 3x = 51	Selected from ‘Longquan No. 1’
6	B352	2n = 3x = 51	Selected from ‘Longquan No. 1’
7	B353	2n = 3x = 51	Selected from ‘Longquan No. 1’
8	B356	2n = 3x = 51	Selected from ‘Longquan No. 1’
9	B431	2n = 4 x = 68	Selected from ‘Longquan No. 1’
10	B456	2n = 4x = 68	Selected from ‘Longquan No. 1’
11	B460	2n = 4x = 68	Selected from ‘Longquan No. 1’
12	B432	2n = 4x = 68	Selected from ‘Longquan No. 1’
13	‘Ruantiaobaisha’	2n = 2x = 34	Zhejiang, China
14	‘Wuheguoyu’	2n = 3x = 51	Selected from ‘Ruantiaobaisha’
15	H424	2n = 4x = 68	Selected from ‘Ruantiaobaisha’
16	H39 [9]	2n = 2x + 5 = 39	Selected from ‘Wuheguoyu’
17	77-1	2n = 2x = 34	Sichuan, China
18	K474	2n = 4x = 68	Selected from 77-1
19	‘Ninghaibai’	2n = 2x = 34	Zhejiang, China
20	‘Huabai No. 1’	2n = 2x = 34	Selected from ‘Ruantiaobaisha’
21	‘Changbai No. 1’	2n = 2x = 34	Fujian, China
22	Q24	2n = 3x = 51	Selected from ‘Changbai No. 1’
23	‘Huayuwuhe No. 1’	2n = 3x = 51	Selected from ‘Changbai No. 1’

### DNA extraction and examination

Genomic DNA isolation was performed based on the cetyltrimethylammonium bromide method of Roche et al. [26]. After isolation, the quality was checked on a 1.0% agarose gel, and all genomic DNA was diluted to 50 ng/μL for SSR-PCR and SSR-qPCR analysis.

### SSR primers

A total of 209 SSRs derived from loquat (16), pear (42), apple (144), peach (5), apricot (1) and plum (1) were used in this study (Table 2). Some of the SSRs were located in the 17 LGs of bronze loquat [27], and some were also located on genetic linkage maps of the Japanese pears ‘Housui’ or the European pear ‘Bartlett’ and ‘La France’ [28–31]. Almost all loquat LGs were aligned with the pear consensus map by using at least two apple or pear SSRs [27]. The primers were synthesized by Shanghai Invitrogen™ Life Technologies.

**Table 2:** SSR markers used for cross-genus amplification in loquat

SSR name	Origin	Reference
**ssrEJ014**, ssrEJ086, ssrEJ088, ssrEJ042, ssrEJ282, ssrEJ049, **ssrEJ324**, ssrEJ061, ssrEJ104, **ssrEJ329b**, ssrEJ037, ssrEJ066, ssrEJ056, **ssrEJ046**, ssrEJ012, ssrEJ271	Loquat	[35]
**NB105a**, NB103a, NB106a, NB101a, NB114a, **NH033b**, **NH011b**, **NH026a**, NH007b, NH024b, KA16	Pear	[36–38]
**IPPN14**, **IPPN09**	Pear	[39]
**TsuENH094**, **TsuENH003**, TsuENH184, TsuENH119, TsuENH174, TsuENH207, TsuENH229, TsuENH017, **TsuENH074**, **TsuENH044**, **TsuENH069**, **TsuENH086**, **TsuENH067**, **TsuENH034**, TsuENH005, **TsuENH097**, **TsuENH009**, **TsuENH004**, **TsuENH093**, **TsuENH096**, **TsuENH031**, **TsuENH032**, **TsuENH007**, TsuENH022, TsuENH042, **TsuENH002**, TsuENH028, TsuENH033, **TsuENH080**	Pear	[30]
**CH05g08**, **CH02f06**, **CH03d10**, **CH04e12b**, **CH02b10**, CH03d01, CH02c06, CH03b01, **CH03g12**, CH03g07, CH04g07, **CH01d03**, CH01d09, **CH02h11a**, **CH01b12**, CH03a09, CH04f04, **CH04e03**, CH02b12, CH04g09, CH03d07, CH03d12, **CH04e05**, **CH05c02**, CH01f09, CH01h10, CH05a02, **CH05c07**, CH01h02, CH05a03, **CH01f03b**, **CH04c06**, **CH04f03**, CH01f07a, CH02b03b, CH05h12, **CH02d12**, CH04a12, **CH04d07**, CH04h02, **CH04d02**, **CH01f02**, CH04g04, **CH05d04**, **CH05g07**, **CH03c02**, **CH02e02**, **CH02g01**, **CH03a08**, CH03h03, CH05h05, **CH04f06**, **CH05g11**, CH04c07, **CH01d08**, **CH02e12**, CH01a09, CH01e01, CH03g06, CH05d03, **CH02c09**, **CH03h06**, CH05g05, CH01f03a, CH05c06, **CH02a03**, CH05e04, **CH02d10a**, CH05a09, **CH04b10**, CH04f10, **CH05a04**, **CH04c10**, CH05g03, **CH01h01**, **CH01c08**, CH04g12, CH02h11b, **CH05d11**, MS14h03, MS06c09, MS06g03, MS14b04	Apple	[40]
**AJ320188SSR**, AT000400SSR, **AT000174SSR**, AU223657SSR, **AU223548SSR**, AY187627SSR, **CN581493SSR**, **CN493139SSR**, **CN444542SSR**, Hi02c07, **Hi03e04**, Hi07e08, Hi15h12, Hi04g11, Hi08a04, **Hi03a03**, Hi04a05, **Hi05b02**, **Hi08h12**, Hi04g05, Hi15a13, **Hi22f06**, **Hi04e04**, **Hi07h02**, **Hi23d11b**, **U78949SSR**, Z71980SSR	Apple	[41]
**NZ04f3**, NZ02b1	Apple	[42]
NZmsCN879773, NZmsEB149808, NZmsEB177464, NZmsEB155242, **NZmsEB134379**, **NZmsCN898349**, **NZmsCO754252**, **NZmsCN943067**, **NZmsEB137749**, **NZmsDR033893**, **NZmsEB111793**, **NZmsCN878021**, **NZmsEE663955**	Apple	[43]
**MEST012**, **MEST028**, MEST067, **MEST089**, MEST095, MEST034, MEST063, **MEST023**, MEST038, **MEST043**, **MEST011**, MEST003, MEST041, **MEST029**, MEST069, **MEST096**, **MEST091**	Apple	[44]
GD142	Apple	[45]
CH-Vf1	Apple	[46]
M06a	Peach	[47]
BPPCT030, BPPCT008, BPPCT014, BPPCT006	Peach	[48]
ssrPaCITA16	Apricot	[49]
CPSCT026	Plum	[50]

The stably amplified SSR markers are shown in bold and the SSR markers with polymorphisms are underlined

### PCR amplification and electrophoresis of SSRs

SSR reactions were performed in a final volume of 20 μL, containing 2.0 μL of 10× PCR buffer (Mg^2+^ free), 1.5 mM MgCl_2_, 0.0375 mM each dNTP, 0.25 µM each of the forward and reverse primer, 50 ng of genomic DNA and 1 unit of Taq DNA polymerase (Takara Biotechnology Company, Dalian, China). The amplification was performed using a C1000 Touch PCR System thermal cycler (Bio-Rad) with the following temperature conditions: 94 °C for 2.5 min, followed by 35 cycles of 94 °C for 30 s, 60 °C for 30 s and 72 °C for 30 s and a final step of 72 °C for 10 min. The PCR products were mixed with 15% loading buffer (36% glycerol, 30 mM ethylenediaminetetraacetic acid (EDTA), and 0.05% bromophenol blue and xylene cyanol). Three microliters of each mixture was loaded into 8% denaturing polyacrylamide gels (7 M urea) in 1× TBE buffer (89 mM Tris–borate, 1 mM EDTA, pH 8.0). The gels were run at 220 W for 50 min and silver stained [32].

### Chromosome preparation

Chromosomes at mitotic metaphase were prepared from root tip materials according to methods reported by Chen et al. [33]. Root tip tissue with a length of approximately 1 cm was immersed in 0.002 mol/L 8-hydroxyquinoline aqueous solution for 4 h and fixed overnight in Carnoy’s solution (methanol: glacial acetic acid = 3:1). The apical meristems were cut into tissue blocks of approximately 1 mm^3^, rinsed in deionized water, and then incubated in mixed enzyme solution containing 3% cellulose (SCR, China) and 0.3% pectinase (Yakult, Japan). After 3 h, the enzyme solution was removed, the apical meristems were soaked in deionized water for 10 min, and placed in Carnoy’s solution. At last, apical meristems were dispersed on slides with tweezers and dried over the alcohol burner flame. Chromosomes were colorized with 5% Giemsa staining, dried in air, and visualized under a microscope (Olympus, Tokyo, Japan).

### qPCR

qPCR was performed using a StepOne real-time PCR instrument (ABI Corporation). The PCR amplifications were performed in a 10 μL reaction system containing genomic DNA (50 ng), 0.2 µM each primer, corresponding ROX Reference Dye I (0.2 μL), and 2 × NovoStart® SYBR qPCR SuperMix Plus (Novoprotein Scientific Inc.) (5 μL). The PCR began at 95 °C for 1 min and then progressed to 40 cycles of 95 °C for 20 s and 60 °C for 1 min. This programme was followed by 95 °C for 15 s and 60 °C for 1 min. The fluorescence was measured at 60 °C for 40 cycles.

### Data analysis

We first obtained the PCR product amount (ΔRn value) ratios of the 17 LGs in known euploid strains as controls. Known aneuploid strains and unknown loquat strains were then examined and compared with the controls. A limited number of ΔRn values were expected according to the following aneuploidy and euploidy ΔRn scale: 1 (2x, 3x, and 4x karyotypes), 0.5 (2x − 1 karyotype), 1.5 (2x + 1 karyotype), 0.75 (4x − 1 karyotype) and 1.25 (4x + 1 karyotype).

StepOne v2.1 software (ABI Corporation) was used to calculate the ΔRn values of each segmental duplication. Each LG had one SSR marker, and the fluorescence intensity of each SSR marker in a single material was calculated. The ΔRn values were calculated based on the method of Zimmermann et al. [34]. To the extent possible, parallel amplification curves were obtained during the exponential growth period to ensure equal amplification of the 17 sites. The ΔRn values with the cycle threshold (CT) values between 16 and 24 were selected, and the ΔRn ratios were examined at three separate points (bottom, middle and top) along the amplification curve in the exponential phase. Samples that deviated were disregarded. All ΔRn values of the SSR markers were standardized with the ΔRn of NZmsCO754252 as the reference standard. The proportions of ΔRn values for whole chromosomes were used to discriminate aneuploid individuals and euploid individuals. Analysis of variance was performed by using SPSS 19.0.

## Results

### SSR analysis

Our goal was to obtain 17 amplified stable SSR markers covering all loquat LGs. Of all the 209 SSR markers derived from loquat, pear, apple, peach, apricot and plum, 102 SSRs (approximately half) were applicable to loquat, of which 28 (approximately 13.4% of the total) showed polymorphism (Table 2).

### Screening of 17 SSR markers covering all loquat LGs by qPCR

We aimed to develop a rapid and reliable method for studying aneuploid karyotypic abnormalities. For the detection of aneuploidy, 17 SSR markers were selected from a previously published high-density genetic linkage map [27], and primers for 6 of the 17 SSR markers were redesigned according to the expressed sequences, with at least one well-conserved locus from each LG (Additional file 1: Fig. S1). These 17 SSR markers were amplified with the same efficiency during the exponential phase of PCR and used as target sequences to detect the number of chromosomes in each LG. Table 3 presents the primer sequences and product lengths.

**Table 3:** Primer information for 17 SSRs

SSR name	Primer sequences (5′ → 3′)	Origin	LG in loquat	Product size/bp	T (°C)
TsuENH094	F: GAAGAAGCAAAACCCGAAGA R: TTGTTCTCCTCGCCACCTT	Pear	LG1	155	60
MEST028	F: ATTGGCATTGCTTCTCACC R: TGCAACAACAATTCCCTTCA	Apple	LG2	148	60
CH03g12	F: GCGCTGAAAAAGGTCAGTTT R: CAAGGATGCGCATGTATTTG	Apple	LG3	154–200	60
TsuENH044	F: TGGCTAAATACTCTTCTCGAAAACAA R: GTGATTATTATAGATACCAAGCCTCTC	Pear	LG4	127	60
NZmsCN898349	F: GAGTTGGCAGAAAGAAACCA R: CTGGGTGAAGACGAGATGCT	Apple	LG5	200	60
NZmsCO754252	F: CTGCCCTCAAGGAGAATGTC R: ACAGGTGCAGCAAAGGCTAT	Apple	LG6	195	60
NZmsEB137749	F: ATCTCCTGCTGTGCTGGTCT R: TCACCAAACACCAATCAACAA	Apple	LG7	220	60
TsuENH034	F: CATTATCCATTTGATTAAACTACACG R: GGTAGAAAGAGAAGGAAAGTGGG	Pear	LG8	151	60
TsuENH097	F: CTGACACCCACTACGATTCAAGA R: AAACGAGCTTGGTACGGATTACA	Pear	LG9	162	60
Hi05b02	F: GATGCGGTTTGACTTGCTTC R: GTTTCTCCAGCTCCCATAGATTGC	Apple	LG10	120–178	60
IPPN14	F: GAGGAAGTAACCGCATCAGC R: TCTAAGGGCAGGCAGATCAC	Pear	LG11	223–252	60
MEST011	F: GCGTGAGTTGAGCAAGATGG R: TAGAAGCAATAAGGTGGAGTGGT	Apple	LG12	205	60
CH02e02	F: CTCATCAGTCTCACTGACTGTGTG R: AGGGTCAGGGTCAGTCAGG	Apple	LG13	122–130	60
TsuENH093	F: AGACTGCTGAGGGAATCCATAA R: TTCCGAGTCAAATGGGGC	Pear	LG14	144	60
TsuENH007	F: ATTCATTGCACCGACTACCGATT R: AGTGGCGTAGTGGGAAGGG	Pear	LG15	166	60
Hi22f06	F: CAATGGCGTCTGTGTCACTC R: GTTTACGACGGGTAAGGTGATGTC	Apple	LG16	240–246	60
TsuENH002	F: CAGCAGGAAACACAGAAAAACAG R: ATATCGAGCAATCAAGGAAGCAG	Pear	LG17	116	60

The primers for TsuENH094, TsuENH044, TsuENH034, MEST011, TsuENH093, and TsuENH007 were designed by the authors based on the expressed sequences (National Center for Biotechnology Information, NCBI). The information on the LGs in loquat was obtained from Fukuda et al. [27]

After qPCR detection, each of the dissolution curves of the 17 pairs of SSR primers showed a single peak (Additional file 2: Fig. S2). The CT values of the 17 pairs of SSR primers were between 15 and 25, and the negative control had no CT value, which indicated that the PCR primer was specific.

### Detection of molecular karyotype abnormalities in aneuploid loquat H39 by SSR-qPCR

We included some known euploid strains, such as ‘Ruantiaobaisha’, ‘Wuheguoyu’ and H424, as controls in the detection of aneuploid loquat H39 (2n = 2x + 5 = 39) karyotype variation. The means and standard deviations of ΔRn of the bottom, middle and top of the exponential phase are shown in Table 4. The results showed that the relative intensities of ΔRn values of the 17 SSR markers were similar among ‘Ruantiaobaisha’, ‘Wuheguoyu’ and H424, whereas the ΔRn values of H39 were altered in five LGs. The ΔRn values obtained for H39 in LG3, LG8, LG10, LG16 and LG17 were approximately 41.8% higher than those obtained for the euploids. The results implied that LG3, LG8, LG10, LG16 and LG17 have one more chromosome in H39 than in the diploid ‘Ruantiaobaisha’. In other words, H39 might have five more chromosomes than the diploid ‘Ruantiaobaisha’. This result was consistent with the result obtained with conventional cytological methods [9]. The statistical means and standard deviations of the ΔRn values obtained for the 17 loquat LGs are shown in Table 4.

**Table 4:** ΔRn values obtained for in four known loquat strains using 17 pairs of SSR primers

SSR name	LG in loquat	‘Ruantiaobaisha’	‘Wuheguoyu’	H424	Mean control	H39
TsuENH094	LG1	0.96 ± 0.02	0.97 ± 0.01	0.94 ± 0.02	0.96 ± 0.02	0.96 ± 0.02
MEST028	LG2	0.96 ± 0.06	0.95 ± 0.05	0.94 ± 0.03	0.95 ± 0.01	0.98 ± 0.05
CH03g12	LG3	0.98 ± 0.04	0.91 ± 0.03	0.97 ± 0.05	0.95 ± 0.04	1.42* ± 0.03
TsuENH044	LG4	0.99 ± 0.01	0.95 ± 0.20	1.01 ± 0.04	0.98 ± 0.03	1.08 ± 0.15
NZmsCN898349	LG5	1.03 ± 0.03	1.01 ± 0.04	1.00 ± 0.02	1.01 ± 0.02	1.01 ± 0.03
NZmsCO754252	LG6	1.00 ± 0.03	1.00 ± 0.03	1.00 ± 0.01	1.00 ± 0.00	1.00 ± 0.01
NZmsEB137749	LG7	1.04 ± 0.00	0.98 ± 0.09	1.01 ± 0.02	1.01 ± 0.03	0.99 ± 0.01
TsuENH034	LG8	0.98 ± 0.01	0.99 ± 0.01	0.98 ± 0.01	0.98 ± 0.01	1.35* ± 0.07
TsuENH097	LG9	1.02 ± 0.02	1.02 ± 0.01	1.00 ± 0.01	1.01 ± 0.01	0.96 ± 0.00
Hi05b02	LG10	1.00 ± 0.04	1.01 ± 0.05	0.99 ± 0.03	1.00 ± 0.01	1.31* ± 0.06
IPPN14	LG11	1.04 ± 0.03	1.04 ± 0.03	1.02 ± 0.01	1.03 ± 0.01	0.95 ± 0.02
MEST011	LG12	1.04 ± 0.05	1.08 ± 0.05	0.98 ± 0.04	1.03 ± 0.05	0.93 ± 0.10
CH02e02	LG13	1.03 ± 0.04	0.99 ± 0.02	0.99 ± 0.05	1.00 ± 0.02	0.94 ± 0.04
TsuENH093	LG14	1.04 ± 0.02	1.03 ± 0.01	1.02 ± 0.02	1.03 ± 0.01	1.00 ± 0.02
TsuENH007	LG15	1.07 ± 0.00	0.98 ± 0.04	0.98 ± 0.01	1.01 ± 0.05	0.97 ± 0.02
Hi22f06	LG16	1.11 ± 0.02	1.07 ± 0.02	0.95 ± 0.07	1.04 ± 0.08	1.51* ± 0.03
TsuENH002	LG17	1.11 ± 0.12	1.08 ± 0.09	0.99 ± 0.08	1.06 ± 0.06	1.50* ± 0.03

The SSR-qPCR results are expressed as the means ± standard deviations, * indicates LGs with abnormalities in the chromosome dosage

### Application of molecular karyotypes to triploid offspring populations

**Fig. 2:**
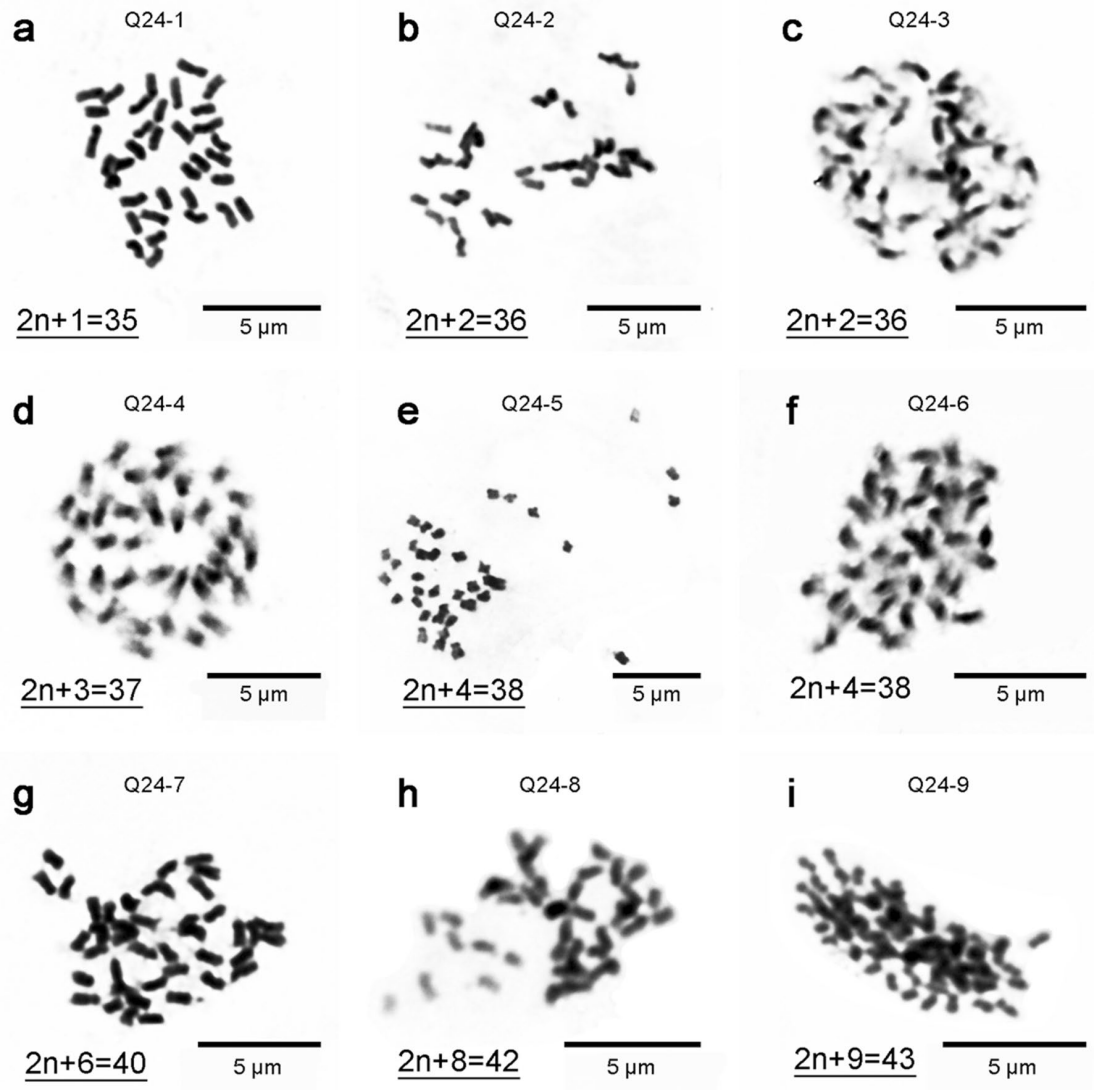
Mitotic metaphase chromosomes of 9 hybrid offspring of Q24× ‘Huabai No. 1’. The strain name of each chromosome preparation corresponds to that in the genetic diagram shown in Fig. 1, and the underlined chromosome numbers are consistent with the SSR-qPCR results

**Fig. 3:**
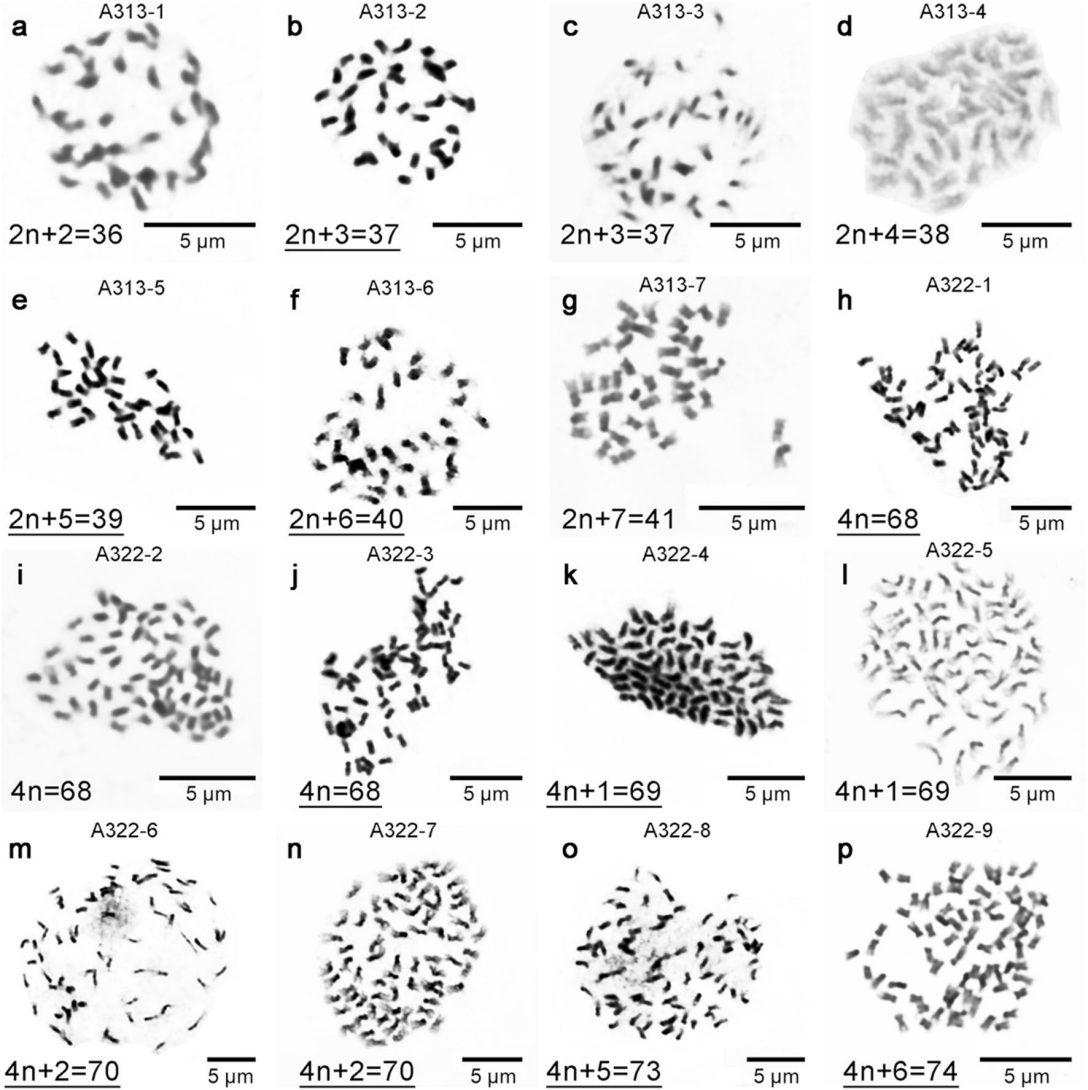
Mitotic metaphase chromosomes of 16 open-pollination progeny of triploid loquat strains (A313 and A322). The strain name of each chromosome preparation corresponds to that in the genetic diagram shown in Fig. 1, and the underlined chromosome numbers are consistent with the SSR-qPCR results

To verify the method for the detection of aneuploid karyotype variation, 9 hybrid offspring of Q24× ‘Huabai No. 1’, 7 open-pollination progeny of the triploid loquat A313 and 9 open-pollination progeny of the triploid loquat A322 were obtained, and these included a total of 22 aneuploids and 3 euploids. We detected their chromosome numbers using conventional cytological methods (Table 5, Figs. 2, 3).

**Table 5:** Numbers of chromosomes in 9 hybrid offspring of Q24 x ‘Huabai No. 1’ and 16 open-pollination progeny of triploid loquat strains (A313 and A322)

Female parent	2n = 2x + 1 = 35	2n = 2x + 2 = 36	2n = 2x + 3 = 37	2n = 2x + 4 = 38	2n = 2x + 5 = 39	2n = 2x + 6 = 40	2n = 2x + 7 = 41	2n = 2x + 8 = 42	2n = 2x + 9 = 43	2n = 4x = 68	2n = 4x + 1 = 69	2n = 4x + 2 = 70	2n = 4x + 5 = 73	2n = 4x +6 = 74
Q24	1	2	1	2		1		1	1					
A313		1	2	1	1	1	1							
A322										3	2	2	1	1
Total	1	3	3	3	1	2	1	1	1	3	2	2	1	1

To quantify the agreement rate between the SSR-qPCR and conventional cytological methods, the SSR-qPCR method was used to estimate and describe the level of aneuploidy in the 9 hybrid offspring of Q24× ‘Huabai No. 1’ (Additional file 3: Table S2). We included some known euploid strains, such as ‘Huabai No. 1’, ‘Changbai No. 1’, Q24 and ‘Huayuwuhe No. 1’ (Additional file 3: Table S1) as controls in the SSR-qPCR analysis of the detection of aneuploid molecular karyotype variation in the offspring of Q24× ‘Huabai No. 1’. The detection results showed that the ΔRn values of the control strains were between 0.84 and − 1.08, and 88.9% of the SSR-qPCR results for the 9 hybrids, including 8 trisomic samples, were consistent with the results obtained using conventional cytological methods. Furthermore, the LGs of the added chromosomes in 8 loquat aneuploids were located (Fig. 2a–e, g–i; Table 6). Eight trisomic samples were detected to have trisomy mainly in LG1, LG2, LG7, LG10, LG13, and LG14.

**Table 6:** SSR-qPCR results for 23 known strains and 25 unknown triploid offspring strains

Strain name	Pedigree	Ploidy	SSR-qPCR result	LG of abnormal chromosome
‘Dawuxing’	NK	2n = 2x = 34	Euploidy	NA
A313	Selected from ‘Dawuxing’ (2x)	2n = 3x = 51	Euploidy	NA
A322	Selected from ‘Dawuxing’ (2x)	2n = 3x = 51	Euploidy	NA
‘Longquan No. 1’	NK	2n = 2x = 34	Euploidy	NA
B350	Selected from ‘Longquan No. 1’ (2x)	2n = 3x = 51	Euploidy	NA
B352	Selected from ‘Longquan No. 1’ (2x)	2n = 3x = 51	Euploidy	NA
B353	Selected from ‘Longquan No. 1’ (2x)	2n = 3x = 51	Euploidy	NA
B356	Selected from ‘Longquan No. 1’ (2x)	2n = 3x = 51	Euploidy	NA
B431	Selected from ‘Longquan No. 1’ (2x)	2n = 4x = 68	Euploidy	NA
B456	Selected from ‘Longquan No. 1’ (2x)	2n = 4x = 68	Euploidy	NA
B460	Selected from ‘Longquan No. 1’ (2x)	2n = 4x = 68	Euploidy	NA
B432	Selected from ‘Longquan No. 1’ (2x)	2n = 4x = 68	Euploidy	NA
‘Ruantiaobaisha’	NK	2n = 2x = 34	Euploidy	NA
‘Wuheguoyu’	Selected from ‘Ruantiaobaisha’ (2x)	2n = 3x = 51	Euploidy	NA
H424	Selected from ‘Ruantiaobaisha’ (2x)	2n = 4x = 68	Euploidy	NA
H39	Selected from ‘Wuheguoyu’ (3x)	2n = 2x + 5 = 39	2n = 2x + 5 = 39	LG3, LG8, LG10, LG16, LG17
77-1	NK	2n = 2x = 34	Euploidy	NA
K474	Selected from 77-1 (2x)	2n = 4x = 68	Euploidy	NA
‘Ninghaibai’	NK	2n = 2x = 34	Euploidy	NA
‘Huabai No. 1’	Selected from ‘Ruantiaobaisha’ (2x)	2n = 2x = 34	Euploidy	NA
‘Changbai No. 1’	NK	2n = 2x = 34	Euploidy	NA
Q24	Selected from ‘Changbai No. 1’ (2x)	2n = 3x = 51	Euploidy	NA
‘Huayuwuhe No. 1’	Selected from ‘Changbai No. 1’ (2x)	2n = 3x = 51	Euploidy	NA
Q24-1	Q24 (3x)×‘Huabai No. 1’ (2x)	2n = 2x + 1 = 35	2n = 2x + 1 = 35	LG16
Q24-2	Q24 (3x)×‘Huabai No. 1’ (2x)	2n = 2x + 2 = 36	2n = 2x + 2 = 36	LG1, LG17
Q24-3	Q24 (3x)×‘Huabai No. 1’ (2x)	2n = 2x + 2 = 36	2n = 2x + 2 = 36	LG1, LG7
Q24-4	Q24 (3x)×‘Huabai No. 1’ (2x)	2n = 2x + 3 = 37	2n = 2x + 3 = 37	LG2, LG9, LG15
Q24-5	Q24 (3x)×‘Huabai No. 1’ (2x)	2n = 2x + 4 = 38	2n = 2x + 4 = 38	LG2, LG6, LG8, LG10
Q24-6	Q24 (3x)×‘Huabai No. 1’ (2x)	2n = 2x + 4 = 38	Failed to detect	Failed to detect
Q24-7	Q24 (3x)×‘Huabai No. 1’ (2x)	2n = 2x + 6 = 40	2n = 2x + 6 = 40	LG10, LG12, LG13, LG14, LG15, LG16
Q24-8	Q24 (3x)×‘Huabai No. 1’ (2x)	2n = 2x + 8 = 42	2n = 2x + 8 = 42	LG2, LG3, LG5, LG7, LG9, LG10, LG13, LG14
Q24-9	Q24 (3x)×‘Huabai No. 1’ (2x)	2n = 2x + 9 = 43	2n = 2x + 9 = 43	LG1, LG4, LG7, LG10, LG11, LG12, LG13, LG14, LG17
A313-1	Selected from A313 (3x)	2n = 2x + 2 = 36	Failed to detect	Failed to detect
A313-2	Selected from A313 (3x)	2n = 2x + 3 = 37	2n = 2x + 3 = 37	LG1, LG7, LG14
A313-3	Selected from A313 (3x)	2n = 2x + 3 = 37	Failed to detect	Failed to detect
A313-4	Selected from A313 (3x)	2n = 2x + 4 = 38	Failed to detect	Failed to detect
A313-5	Selected from A313 (3x)	2n = 2x + 5 = 39	2n = 2x + 5 = 39	LG1, LG7, LG9, LG10, LG12
A313-6	Selected from A313 (3x)	2n = 2x + 6 = 40	2n = 2x + 6 = 40	LG1, LG4, LG7, LG11, LG12, LG14
A313-7	Selected from A313 (3x)	2n = 2x + 7 = 41	Failed to detect	Failed to detect
A322-1	Selected from A322 (3x)	2n = 4x = 68	Euploidy	NA
A322-2	Selected from A322 (3x)	2n = 4x = 68	Failed to detect	Failed to detect
A322-3	Selected from A322 (3x)	2n = 4x = 68	Euploidy	NA
A322-4	Selected from A322 (3x)	2n = 4x + 1 = 69	2n = 4x + 1 = 69	LG12
A322-5	Selected from A322 (3x)	2n = 4x + 1 = 69	Failed to detect	Failed to detect
A322-6	Selected from A322 (3x)	2n = 4x + 2 = 70	2n = 4x + 2 = 70	LG4, LG8
A322-7	Selected from A322 (3x)	2n = 4x + 2 = 70	2n = 4x + 2 = 70	LG8, LG9
A322-8	Selected from A322 (3x)	2n = 4x + 5 = 73	2n = 4x + 5 = 73	LG1, LG5, LG7, LG13, LG14
A322-9	Selected from A322 (3x)	2n = 4x + 6 = 74	2n = 4x + 6 = 74	LG1, LG3, LG5, LG12, LG14, LG17

*NK *not known, *NA* no abnormality

The SSR-qPCR method was also used to estimate and describe the level of aneuploidy in 16 open-pollination progeny of triploid loquat strains (A313 and A322) (Additional file 3: Table S3). In the SSR-qPCR analysis of the detection of aneuploid molecular karyotype variation in the offspring of A313 and A322, we included 22 known euploid loquat strains as controls (Additional file 3: Table S1). The detection results showed that the ΔRn values of the control strains were between 0.81 and − 1.15, and 62.5% of the SSR-qPCR results for 16 open-pollination progeny, including 2 tetraploid samples (Fig. 3h, j), 3 trisomic samples (Fig. 3b, e, f) and 5 pentasomic samples (Fig. 3k, m–p), were consistent with the results obtained using conventional cytological methods.

## Discussion

### SSR-qPCR is a fast, convenient and accurate method for the molecular karyotypes of aneuploids

Our results demonstrate that dosage abnormalities of chromosomes and complete molecular karyotypes of loquat aneuploids can be detected by SSR-qPCR. The 17 SSR markers that used in this study were sequentially located in 17 LGs according to a high-density genetic linkage map [27]. We used 17 SSR markers without polymorphisms and detected them by qPCR. The analysis of the relative proportions of the ΔRn values of the 17 SSR markers (Table 4) allowed the detection of 17 loquat LGs and corresponding molecular karyotypes associated with aneuploidy. Aneuploidy-associated dosage abnormalities produce different ratios of the corresponding amplicons, which result in ΔRn values that are notably different from euploid individuals sample. When performing qPCR to diagnose abnormalities of any given chromosomes in samples, SSR markers for other autosomes can be selected as standards [13]. Our method could clearly differentiate changes in the chromosome dosage as low as 1.16-fold for trisomic diagnosis, and the ΔRn values of the 22 known euploid loquat strains were 1.00 ± 0.05. Furthermore, the changes of the chromosome copy number in 17 loquat LGs were identified through assessment of the ΔRn ratios between 17 SSR markers.

Similar to conventional cytological methods, SSR-qPCR has several advantages: reliability, low sample demand, ease of performance, and ability to produce precise data. The PCR analysis can directly generate data for analysis without the need for gel electrophoresis or other processing steps, which saves considerable time. Notably, karyotyping through conventional cytological analysis is difficult for some species, such as *A. thaliana*, due to the lack of chromosome-specific probes and the small sizes of chromosomes [3]. Kiwifruit (2n = 2x = 58) chromosomes are small and numerous, and the average length of each chromosome is 0.6–1.5 μm [51–53]. Liang et al. analysed the karyotypes of 10 diploid varieties of the genus *Malus*, all of which have small chromosomes, and found that the difference between the longest and shortest chromosomes was often very small [54]. These factors make conventional karyotype analyses relatively difficult. During the detection of aneuploid individuals in a polyploid population, the difficulty of classic cytological chromosome counting increases with the number of polyploid chromosomes. Many major cultivars are polyploid, such as allotetraploid cotton (*Gossypium hirsutum* and *Gossypium barbadense*) [55], octoploid strawberry (*Fragaria* × *ananassa*) [56], and the octoploid and decaploid *Actinidia. arguta var. giraldii* [57]. In addition, seedling population experiments do not provide sufficient material for distinguishing chromosomes, and cutting the root tip of a seedling is likely to lead to death; however, these difficulties can be avoided through the use of SSR-qPCR. SSR-qPCR also has other advantages in contrast to conventional cytology: genomic DNA in all organs of an individual is identical at all periods of growth, and the SSR-qPCR technique is not limited to the tips of roots and stems. qPCR using SSR markers based on genomic DNA is more convenient and stable than classic cytology, and SSR markers of 17 LGs were selected in this study as cross-references. The method is convenient and produces data that are easy to analyse. Thus, SSR-qPCR provides a novel alternative for the detection of aneuploid individuals. Of course, SSR-qPCR also has some limitations. For a certain species, we must screen appropriate markers in each LG according to a high-density genetic linkage map based on markers, and unexpected SNPs or copy number variations (CNVs) can lead to errors in judgement. Replacing markers or increasing the number of markers can minimize these errors in judgement.

Similar to microarray-based comparative genomic hybridization (array-CGH), SSR-qPCR can be used to detect changes in fluorescence intensity caused by chromosomal abnormalities, and changes in the chromosome copy number in the whole-genome can be detected through just one experiment. Array-CGH detects abnormalities in the initial chromosome copy number of a sample and requires the use of different fluorescent labels. The method is complicated and extremely expensive in terms of chip cost; therefore, array-CGH is not suitable for large-scale experiments [58]. SSR-qPCR amplifies the fluorescence intensity through PCR amplification of the initial chromosome copy number. All chromosomes are subjected to qPCR using the SYBR Green I dye. The advantages of relatively low cost and simple operation make SSR-qPCR suitable for large-scale experiments. Additionally, when whole-genome sequences are used, the molecular karyotypes obtained by SSR-qPCR and array-CGH correspond to the karyotypes obtained using conventional cytological methods, and the application potential is higher.

Moreover, qPCR directly quantifies the fluorescence intensity, which is directly proportional to the amount of PCR product amplified. The examination of ΔRn values might be more accurate than a method that relies solely on CT values [34]. Therefore, the SSR-qPCR method will yield more accurate and detailed information on molecular karyotypes compared with other methods. In this study, SSR-qPCR was used to construct a molecular karyotype of all chromosomes by detecting the gene dosage balance in the exponential phase (Table 6). In addition, this method allows the simultaneous processing of all LGs of one sample, which will greatly improve the accuracy of the analysis while reducing costs. We calculated the cost of producing a single aneuploid individual karyotype using the SSR-qPCR method to be less than $10.

### SSR-qPCR is suitable for both heterozygous and homozygous alleles

Detection using the SSR-qPCR method is based on the amount of the PCR product amplified. Some studies based on QF-PCR have shown that small changes in the length of the sequences do not affect the amount of the PCR product amplified. For example, the sequence of Chr13 differs from that of Chr6 by 3 bp but this difference does not affect the trisomy detection of Chr13 by QF-PCR [16]. In addition, QF-PCR of 12 loci can be used for the simultaneous detection of aneuploid and polyploid karyotypes of *A. thaliana* [3]. The aneuploidy detection rate in the prenatal diagnosis of 22,504 samples has been determined to be 98.6% using QF-PCR [59]. The peak ratios of capillary electrophoresis in these studies are consistent with the theoretical results. QF-PCR can accurately detect the chromosomes with trisomiy to describe the molecular karyotypes of partial aneuploids and is widely used for prenatal detection in humans [15, 16]. These studies indicate that QF-PCR based on codominant markers is suitable for heterozygous alleles. Detection using both the QF-PCR and SSR-qPCR methods are based on the amount of the PCR product amplified. Similarly, SSR-qPCR can also be applied to heterozygous alleles.

Our method is more suitable for homozygous alleles than heterozygous alleles. The 17 SSR markers used in this study produced single bands and were not polymorphic. We found that these 17 SSR markers were relatively conserved in 23 known loquat strains. However, these 17 SSR markers might not be suitable for some unknown loquat varieties. The genetic heterozygosity of fruit trees is generally high. In this study, the accuracy of SSR-qPCR for 16 open-pollination progeny of triploid loquat strains (A313 and A322) was only 62.5%, whereas the accuracy of SSR-qPCR for 9 hybrid offspring of Q24× ‘Huabai No. 1’ was 88.9%. SSR markers are randomly distributed throughout plant genomes, and the flanking regions of SSR markers are generally relatively conserved single-copy sequences. SSR markers are short tandem repeats. Tandem repeats are usually found in pericentromeres, centromeres or telomeres, and the tandem repeats near centromeres are more conserved than those in other locations [60]. Tandem repeats are suitable cytogenetic markers for molecular karyotyping and chromosome identification [61]. After the loquat genome sequences are published, subsequent studies should select SSR markers located as close to the centromere as possible. Alternatively, it would be equally feasible to exploit other codominant markers without polymorphism on each chromosome.

### SSR-qPCR allows the application of molecular karyotyping to more species

We were able to detect the complete molecular karyotypes of aneuploid individuals using the SSR-qPCR method, which requires a high-density genetic linkage map. SSR markers are codominant, polymorphic, and suitable for the construction of high-density genetic maps [27]. At present, the high-density genetic linkage maps based on SSR markers are available for apple [43], pear [62], loquat [27, 63], grape [64], kiwifruit [65], strawberry [66], papaya [67], longan [68], tomato [69], cucumber [70], spinach [71], cabbage [72], lettuce [73], pepper [74], rape [75], sorghum [76], cranberry [77], tobacco [78], soybean [79, 80], mungbean [81], wheat [82], maize [83], willow [84], orchardgrass [85], oil palm [86], zoysiagrass [87], carnation [88], cotton [89], spruce [90], rice [91], and tall fescue [92]. The studies that produced these maps allow the possibility of constructing complete molecular karyotypes for these species. Whole-genome sequences of apple [93, 94], pear [95, 96], strawberry [97], peach [98], *Prunus mume* [99], sweet orange [100], jujube [101], pineapple [102], and kiwifruit [103], as well as those of rice, maize, wheat, soybean [104], have been published; therefore, we can obtain large numbers of conserved SSR markers located in the centromeres and telomeres from these whole-genome sequences. Therefore, molecular karyotyping using SSR markers and qPCR is a very useful tool for basic genetic research and accurate breeding. Moreover, this method can also be used for euploid populations, in which it is an effective tool for screening aneuploids and measuring the proportion of chromosomal abnormalities at the population level. Additionally, this method involves a substantially shorter operation time and lower assay complexity compared with many conventional methods. The combination of classic cytological karyotyping with molecular karyotyping based on genomic data will drive the development of breeding from the field of cytology to the field of molecular biology.

### Impacts of aneuploidization on plant inheritance and evolution

The impacts of aneuploidization on speciation and evolution have long been ignored. In humans, trisomy is mainly found on chromosomes 21, 18, 13, 11, X, and Y [15, 16, 18]. In *Malus*, aneuploidization of chromosomes mainly occurs in 15 LGs, excluding LG1 and LG8 [12]. We found a new model of aneuploidization in loquat, because trisomies were found in 17 LGs (Table 6). Aneuploidization can result in loquat speciation with both even and odd basic chromosome numbers (Table 5). Aneuploids with extra chromosomes are often used for analyses of chromosomes or genic dosage effects in evolution and genetics studies.

Aneuploidy greatly exceeds euploidy in crosses between a triploid and a diploid parent [105–109]. In this study, 88% of the progeny of triploids were aneuploids (Table 5). Trisomy of the offspring of Q24× ‘Huabai No. 1’ was mainly detected in LG1, LG7, LG10, LG13, and LG14. The regulation of chromosomal gain or loss contributing to aneuploidy might be controlled by different genes; therefore, the effects of the genes on the adaptation and survival of aneuploids might be very different [105, 107, 109]. Spermatozoa in aneuploids and triploids are mostly sterile, but their ova are usually fertile [105, 107, 109–111]. We found that some triploid loquat strains (‘Wuheguoyu’, A313, A322 and Q24) could produce offspring as female parents. Therefore, aneuploids and triploids might be useful as new alternative male-sterile materials in commercial seed crop breeding programmes.

Aneuploidy can initiate special gene expression. For example, dwarfing tree systems, male sterility, multiple petals, tolerance of drought or cold or resistance to disease can result from ‘super-dominant expression’ due to chromosomal gain or ‘pseudo-dominant expression’ due to chromosomal loss in aneuploids [10–12]. Therefore, aneuploidization increases the diversity and breadth of the foundation for natural selection. However, aneuploids are usually less reproductively stable than euploids [105, 107, 109]. Thus, the advantages and disadvantages of aneuploidization should be appropriately evaluated, and their impacts on speciation and evolution should be properly determined.

## Conclusions

In summary, SSR-qPCR can be used to construct molecular karyotypes of loquat aneuploids. This study provides the first demonstration that a strategy using SSR markers and qPCR can be used to successfully describe complete molecular karyotypes of aneuploids. This technique provides a novel alternative for the detection of chromosome aneuploidies. Marker-assisted breeding using SSR markers with stable amplification will effectively accelerate the breeding of loquat aneuploids. The influences of aneuploidization on speciation and evolution have previously been ignored. The greater genetic diversity in aneuploids than in euploids might provide a broader basis for natural selection. Aneuploids and euploids should be more accurately and strongly applied in studies of breeding, genetics and evolutionary biology. This is the first study to provide reliable molecular evidence for aneuploidy in triploid progeny. This study provides a reliable strategy for further exploration of aneuploidy and the enhancement of polyploid breeding programmes for other species.

## Supplementary information

**Additional file 1: Fig. S1.** Seventeen pairs of SSR primers detected in 23 known loquat strains by polyacrylamide gel electrophoresis.

**Additional file 2: Fig. S2.** qPCR melting curves for the 17 pairs of SSR primers.

**Additional file 3: Table S1.** ΔRn values for the 17 pairs of SSR primers in 22 euploid loquat strains. **Table S2.** ΔRn values for the 17 pairs of SSR primers in 9 hybrid offspring of Q24 × ‘Huabai No. 1’. **Table S3.** ΔRn values for the 17 pairs of SSR primers in 16 open-pollination progeny of triploid loquat strains (A313 and A322).

## Data Availability

The datasets supporting the conclusions and a description of the complete protocol are included within the article.

## Abbreviations

array-CGH: Microarray-based comparative genomic hybridization
CNVs: Copy number variations
CT: Cycle threshold
FISH: Fluorescence in situ hybridization
EDTA: Ethylenediaminetetraacetic acid
HRM: High-resolution melting
LG: Linkage group
QF-PCR: Quantitative fluorescent polymerase chain reaction
MLPA: Multiplex ligation-dependent probe amplification
qPCR: Quantitative real-time polymerase chain reaction
ΔRn: Amount of PCR product
SNP: Single nucleotide polymorphism
SSR: Simple sequence repeat
Tm: Melting temperature
