# Supplementary material for: Molecular karyotypes of loquat (Eriobotrya japonica) aneuploids can be detected by using SSR markers combined with quantitative PCR irrespective of heterozygosity
Source: Plant Methods. 2020 Feb 24;16:22. doi: 10.1186/s13007-020-00568-7 (PMC7041098; doi:10.1186/s13007-020-00568-7)
Supplement: Supplementary file 1 — Additional file 1: Fig. S1. Seventeen pairs of SSR primers detected in 23 known loquat strains by polyacrylamide gel electrophoresis. [file 13007_2020_568_MOESM1_ESM.pdf]

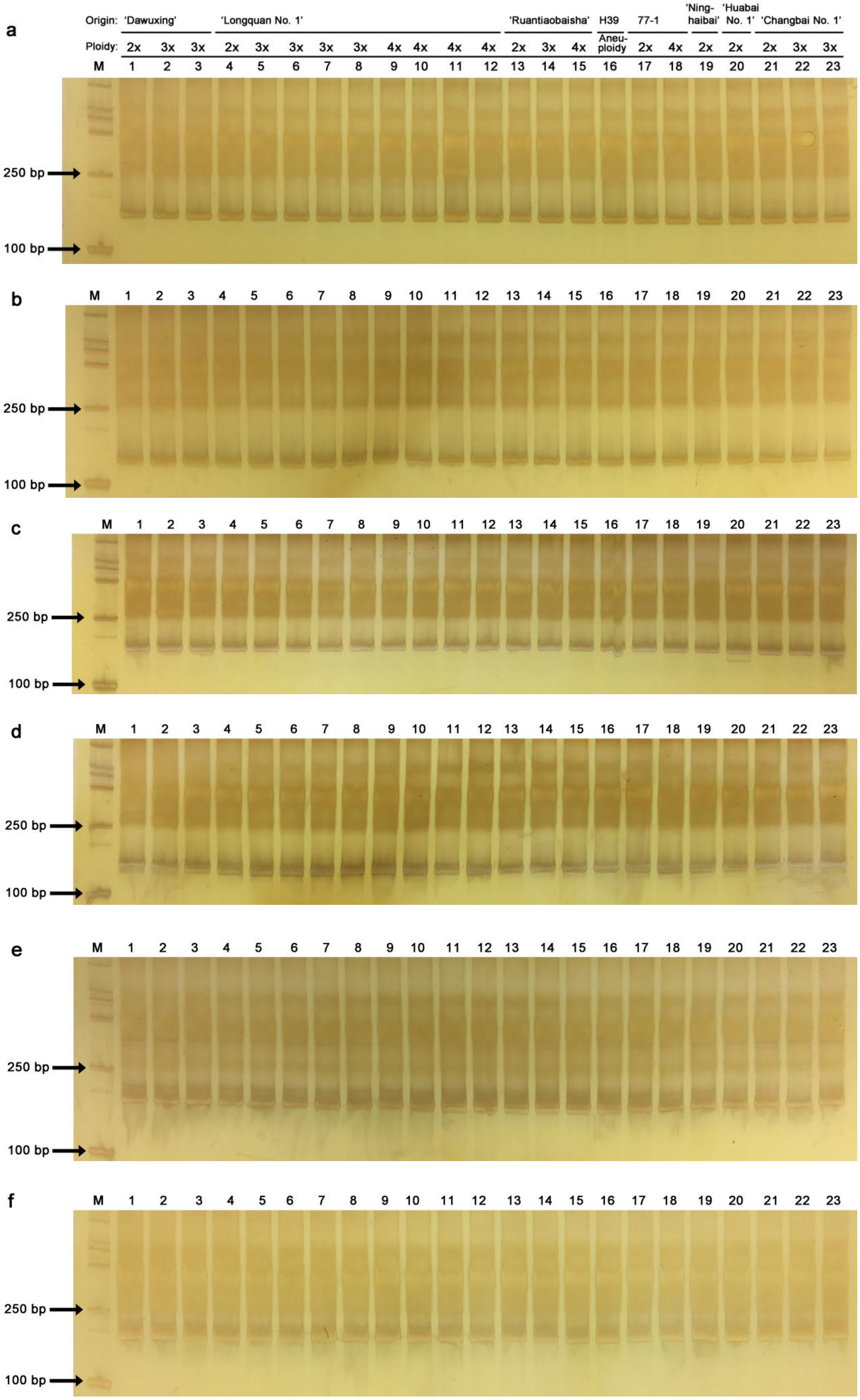
a
Origin: 'Dawuxing' 'Longquan No. 1' 'Ruantiaobaisha' H39 77-1 'Ning-haibai' 'Huabai No. 1' 'Changbai No. 1'
Ploidy: 2x 3x 3x 2x 3x 3x 3x 3x 4x 4x 4x 4x 2x 3x 4x Aneu-ploidy 2x 4x 2x 2x 2x 3x 3x
M 1 2 3 4 5 6 7 8 9 10 11 12 13 14 15 16 17 18 19 20 21 22 23
250 bp
100 bp
b
c
d
e
f

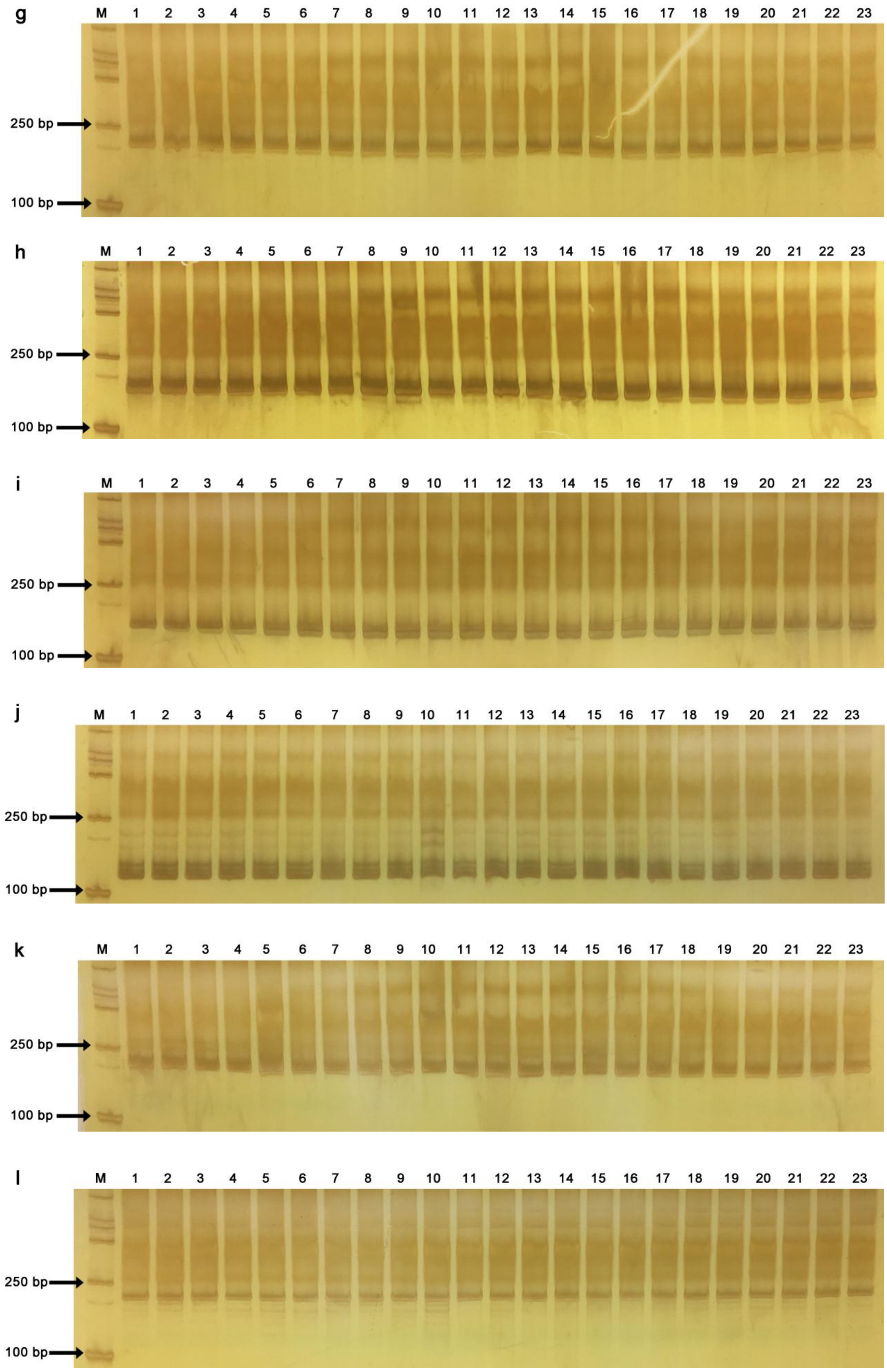
g
M 1 2 3 4 5 6 7 8 9 10 11 12 13 14 15 16 17 18 19 20 21 22 23
250 bp
100 bp
h
M 1 2 3 4 5 6 7 8 9 10 11 12 13 14 15 16 17 18 19 20 21 22 23
250 bp
100 bp
i
M 1 2 3 4 5 6 7 8 9 10 11 12 13 14 15 16 17 18 19 20 21 22 23
250 bp
100 bp
j
M 1 2 3 4 5 6 7 8 9 10 11 12 13 14 15 16 17 18 19 20 21 22 23
250 bp
100 bp
k
M 1 2 3 4 5 6 7 8 9 10 11 12 13 14 15 16 17 18 19 20 21 22 23
250 bp
100 bp
l
M 1 2 3 4 5 6 7 8 9 10 11 12 13 14 15 16 17 18 19 20 21 22 23
250 bp
100 bp

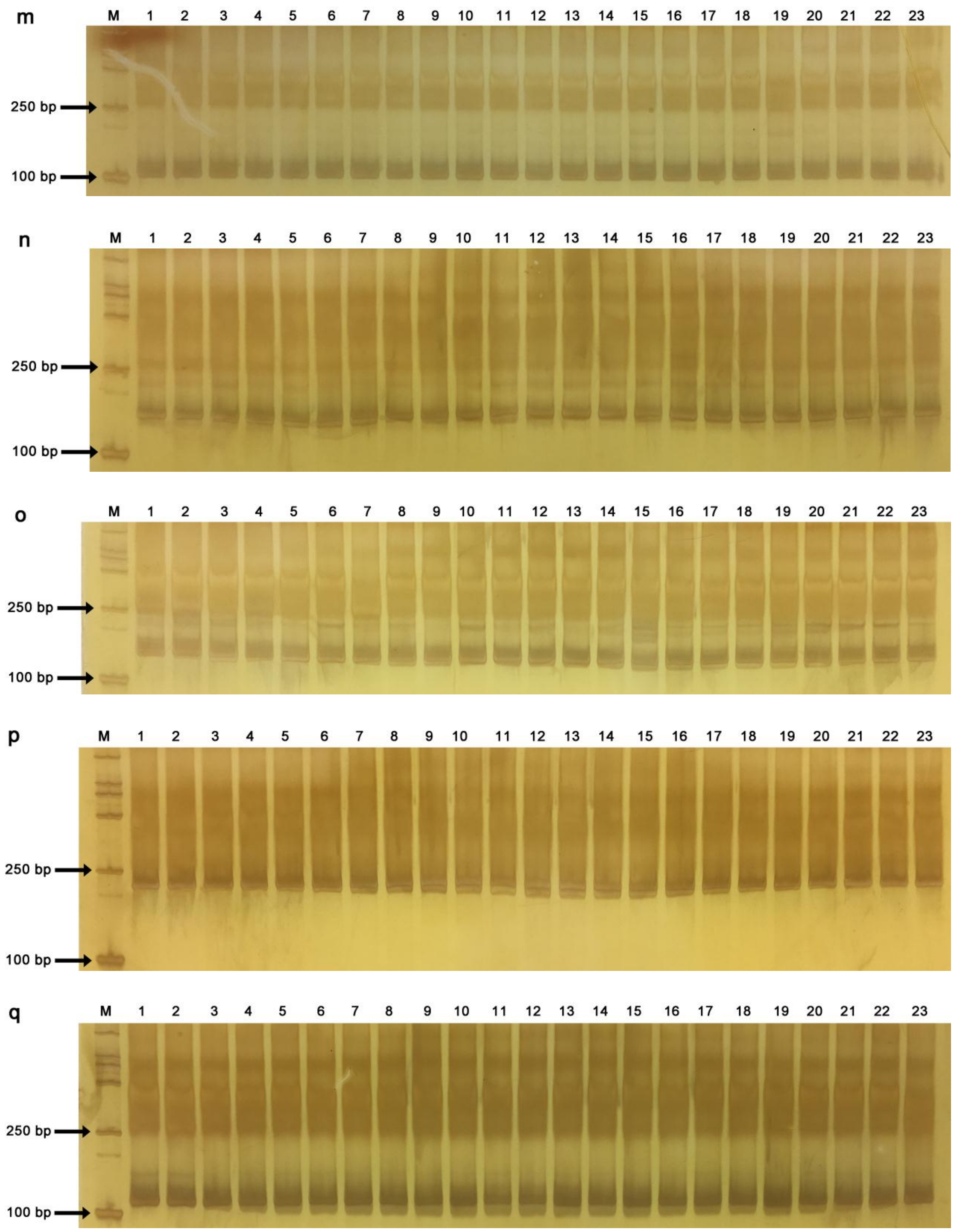

Fig. S1 Seventeen pairs of SSR primers detected in 23 known loquat strains by polyacrylamide gel electrophoresis

M: DL2000 DNA maker. 1-23 sequentially represent 23 known loquat strains used in the experiment. a-q sequentially represent the SSR markers of 17 loquat LGs
