# Supplementary material for: Molecular karyotypes of loquat (Eriobotrya japonica) aneuploids can be detected by using SSR markers combined with quantitative PCR irrespective of heterozygosity
Source: Plant Methods. 2020 Feb 24;16:22. doi: 10.1186/s13007-020-00568-7 (PMC7041098; doi:10.1186/s13007-020-00568-7)
Supplement: Supplementary file 2 — Additional file 2: Fig. S2. qPCR melting curves for the 17 pairs of SSR primers. [file 13007_2020_568_MOESM2_ESM.pdf]

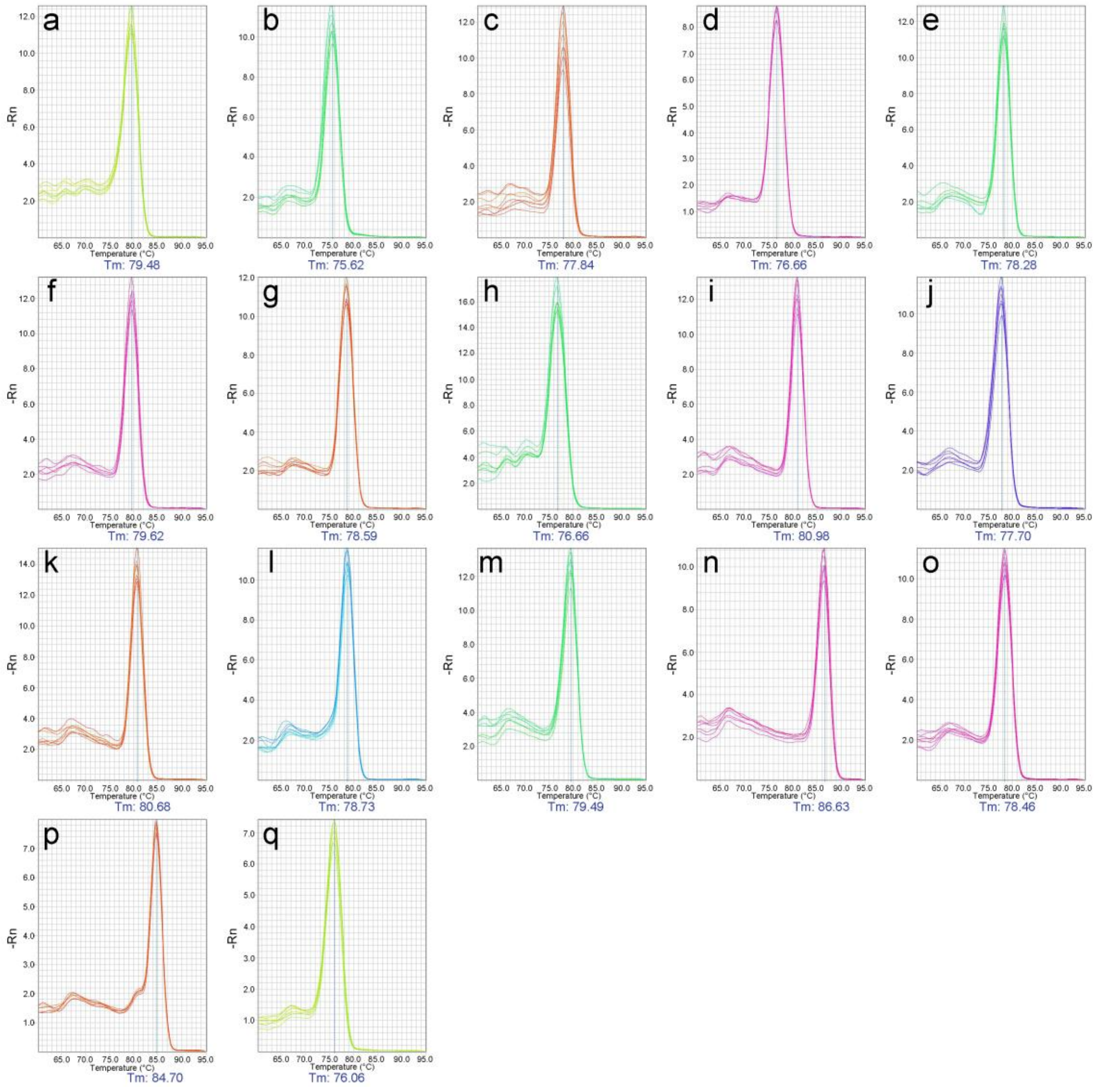

Fig. S2 qPCR melting curves for the 17 pairs of SSR primers

a, TsuENH094; b, MEST028; c, CH03g12; d, TsuENH044; e, NZmsCN898349; f, NZmsCO754252; g, NZmsEB137749; h, TsuENH034; i, TsuENH097; j, Hi05b02; k, IPPN14; l, MEST011; m, CH02e02; n, TsuENH093; o, TsuENH007; p, Hi22f06; q, TsuENH002
