# Supplementary material for: Molecular karyotypes of loquat (Eriobotrya japonica) aneuploids can be detected by using SSR markers combined with quantitative PCR irrespective of heterozygosity
Source: Plant Methods. 2020 Feb 24;16:22. doi: 10.1186/s13007-020-00568-7 (PMC7041098; doi:10.1186/s13007-020-00568-7)
Supplement: Supplementary file 3 — Additional file 3: Table S1. ΔRn values for the 17 pairs of SSR primers in 22 euploid loquat strains. Table S2. ΔRn values for the 17 pairs of SSR primers in 9 hybrid offspring of Q24 × ‘Huabai No. 1’. Table S3. ΔRn values for the 17 pairs of SSR primers in 16 open-pollination progeny of triploid loquat strains (A313 and A322). [file 13007_2020_568_MOESM3_ESM.pdf]

Table S1 ΔRn values for the 17 pairs of SSR primers in 22 euploid loquat strains

SSR name	LG in loquat	'Dawuxing'	A313	A322	'Longquan No. 1'	B350
TsuENH094	LG1	0.92±0.03	1.04±0.04	0.95±0.03	1.04±0.02	1.02±0.03
MEST028	LG2	0.96±0.06	1.10±0.06	0.88±0.01	1.00±0.05	1.00±0.08
CH03g12	LG3	0.99±0.03	1.03±0.03	0.94±0.09	1.01±0.06	0.98±0.05
TsuENH044	LG4	0.87±0.12	0.98±0.14	1.01±0.02	0.96±0.17	0.93±0.08
NZmsCN898349	LG5	1.00±0.04	1.04±0.05	1.01±0.04	0.99±0.08	0.95±0.11
NZmsCO754252	LG6	1.00±0.01	1.00±0.01	1.00±0.02	1.00±0.01	1.00±0.01
NZmsEB137749	LG7	0.95±0.02	1.04±0.03	1.04±0.01	0.96±0.05	0.94±0.06
TsuENH034	LG8	0.86±0.03	1.10±0.03	1.06±0.03	0.86±0.03	1.02±0.06
TsuENH097	LG9	1.02±0.00	0.99±0.07	0.95±0.01	1.04±0.04	1.02±0.01
Hi05b02	LG10	0.98±0.04	1.05±0.01	0.97±0.02	1.02±0.04	1.00±0.04
IPPN14	LG11	0.99±0.01	0.97±0.05	1.02±0.02	0.99±0.04	1.00±0.01
MEST011	LG12	0.94±0.02	1.13±0.07	1.00±0.06	0.95±0.05	1.03±0.04
CH02e02	LG13	1.01±0.03	1.03±0.03	1.01±0.06	1.05±0.06	0.92±0.03
TsuENH093	LG14	0.95±0.03	1.04±0.05	1.00±0.05	1.01±0.00	1.01±0.03
TsuENH007	LG15	1.00±0.02	1.06±0.02	1.00±0.06	1.06±0.01	0.95±0.03
Hi22f06	LG16	1.04±0.01	0.96±0.04	1.07±0.02	1.04±0.02	1.00±0.01
TsuENH002	LG17	0.93±0.10	0.97±0.03	1.06±0.00	0.97±0.09	0.99±0.08

The SSR-qPCR results are expressed as means ± standard deviations

Table S1 (Continued)

SSR name	LG in loquat	B352	B353	B356	B431	B456
TsuENH094	LG1	1.05±0.04	1.01±0.02	1.02±0.08	1.01±0.02	1.07±0.01
MEST028	LG2	1.03±0.04	1.00±0.08	1.04±0.09	1.04±0.03	0.99±0.12
CH03g12	LG3	1.01±0.04	1.05±0.04	1.05±0.06	1.02±0.01	1.02±0.02
TsuENH044	LG4	0.94±0.12	0.99±0.07	1.01±0.12	1.02±0.10	1.11±0.10
NZmsCN898349	LG5	0.99±0.04	0.91±0.13	1.06±0.04	0.98±0.15	0.95±0.12
NZmsCO754252	LG6	1.00±0.02	1.00±0.02	1.00±0.03	1.00±0.04	1.00±0.03
NZmsEB137749	LG7	1.00±0.02	1.03±0.01	1.02±0.03	1.01±0.02	1.00±0.03
TsuENH034	LG8	1.04±0.06	1.07±0.04	0.88±0.02	0.97±0.04	1.04±0.02
TsuENH097	LG9	0.95±0.01	0.99±0.01	1.07±0.01	1.10±0.02	1.04±0.02
Hi05b02	LG10	0.93±0.02	1.01±0.04	1.03±0.04	1.03±0.05	1.03±0.04
IPPN14	LG11	0.95±0.05	0.97±0.00	0.98±0.01	0.97±0.00	1.00±0.01
MEST011	LG12	1.03±0.05	0.93±0.05	0.96±0.07	0.98±0.03	1.04±0.04
CH02e02	LG13	0.90±0.03	0.98±0.06	1.14±0.06	1.04±0.04	1.07±0.04
TsuENH093	LG14	0.96±0.03	1.03±0.01	0.98±0.01	1.04±0.02	0.98±0.03
TsuENH007	LG15	0.99±0.00	0.99±0.01	0.95±0.00	1.04±0.01	1.02±0.01
Hi22f06	LG16	0.94±0.01	0.93±0.05	0.96±0.05	1.09±0.05	0.98±0.11
TsuENH002	LG17	0.99±0.12	1.09±0.10	1.02±0.01	0.89±0.01	1.09±0.07

The SSR-qPCR results are expressed as means ± standard deviations

Table S1 (Continued)

SSR name	LG in loquat	B460	B432	‘Ruantiaobaisha’	‘Wuheguoyu’	H424
TsuENH094	LG1	1.07±0.05	1.01±0.01	0.96±0.02	0.97±0.01	0.94±0.02
MEST028	LG2	1.02±0.07	1.00±0.07	0.96±0.06	0.95±0.05	0.94±0.03
CH03g12	LG3	1.00±0.05	0.99±0.03	0.98±0.04	0.91±0.03	0.97±0.05
TsuENH044	LG4	1.09±0.11	0.90±0.08	0.99±0.01	0.95±0.20	1.01±0.04
NZmsCN898349	LG5	1.00±0.04	0.98±0.11	1.03±0.03	1.01±0.04	1.00±0.02
NZmsCO754252	LG6	1.00±0.02	1.00±0.02	1.00±0.03	1.00±0.03	1.00±0.01
NZmsEB137749	LG7	1.03±0.02	0.96±0.04	1.04±0.00	0.98±0.09	1.01±0.02
TsuENH034	LG8	0.96±0.08	0.92±0.03	0.98±0.01	0.99±0.01	0.98±0.01
TsuENH097	LG9	1.04±0.04	1.07±0.01	1.02±0.02	1.02±0.01	1.00±0.01
Hi05b02	LG10	1.00±0.04	1.02±0.03	1.00±0.04	1.01±0.05	0.99±0.03
IPPN14	LG11	0.98±0.01	1.04±0.02	1.04±0.03	1.04±0.03	1.02±0.01
MEST011	LG12	1.02±0.05	1.02±0.05	1.04±0.05	1.08±0.05	0.98±0.04
CH02e02	LG13	1.06±0.05	1.00±0.03	1.03±0.04	0.99±0.02	0.99±0.05
TsuENH093	LG14	1.06±0.04	0.99±0.02	1.04±0.02	1.03±0.01	1.02±0.02
TsuENH007	LG15	1.02±0.02	1.02±0.02	1.07±0.00	0.98±0.04	0.98±0.01
Hi22f06	LG16	1.03±0.04	1.01±0.00	1.11±0.02	1.07±0.02	0.95±0.07
TsuENH002	LG17	1.07±0.03	0.88±0.08	1.11±0.12	1.08±0.09	0.99±0.08

The SSR-qPCR results are expressed as means ±standard deviations

Table S1 (Continued)

SSR name	LG in loquat	77-1	K474	‘Ninghaibai’	‘Huabai No. 1’	‘Changbai No. 1’
TsuENH094	LG1	1.07±0.01	1.04±0.03	1.11±0.01	0.89±0.16	0.96±0.06
MEST028	LG2	1.13±0.07	1.08±0.06	1.09±0.04	0.93±0.05	1.03±0.05
CH03g12	LG3	1.04±0.02	1.02±0.04	1.09±0.05	0.95±0.04	1.01±0.04
TsuENH044	LG4	0.91±0.05	1.09±0.06	1.10±0.15	1.07±0.07	1.02±0.10
NZmsCN898349	LG5	0.96±0.13	1.04±0.07	1.04±0.09	1.00±0.03	0.99±0.04
NZmsCO754252	LG6	1.00±0.00	1.00±0.03	1.00±0.02	1.00±0.02	1.00±0.02
NZmsEB137749	LG7	0.96±0.02	0.94±0.02	1.07±0.02	1.00±0.00	0.97±0.03
TsuENH034	LG8	1.12±0.01	1.07±0.04	1.15±0.03	1.00±0.03	1.06±0.03
TsuENH097	LG9	1.06±0.04	1.07±0.01	0.93±0.03	0.97±0.03	0.94±0.02
Hi05b02	LG10	1.14±0.06	1.08±0.07	1.00±0.03	0.94±0.03	0.92±0.03
IPPN14	LG11	1.01±0.02	1.08±0.01	1.06±0.03	0.98±0.00	0.97±0.00
MEST011	LG12	1.02±0.04	1.06±0.05	1.01±0.04	0.99±0.04	0.99±0.04
CH02e02	LG13	1.10±0.04	0.96±0.05	0.98±0.04	0.92±0.02	0.95±0.03
TsuENH093	LG14	1.04±0.03	0.98±0.03	1.06±0.03	0.95±0.01	0.99±0.02
TsuENH007	LG15	1.04±0.01	0.91±0.01	1.06±0.05	0.98±0.01	0.93±0.03
Hi22f06	LG16	0.81±0.01	0.92±0.02	1.09±0.05	1.08±0.01	0.84±0.01
TsuENH002	LG17	1.04±0.00	0.97±0.09	1.03±0.16	0.95±0.02	1.05±0.12

The SSR-qPCR results are expressed as means ±standard deviations

Table S1 (Continued)

SSR name	LG in loquat	Q24	'Huayuwuhe No. 1'
TsuENH094	LG1	0.88±0.12	0.94±0.14
MEST028	LG2	0.93±0.03	0.95±0.04
CH03g12	LG3	0.97±0.03	0.98±0.05
TsuENH044	LG4	1.00±0.01	1.08±0.02
NZmsCN898349	LG5	1.02±0.04	1.00±0.04
NZmsCO754252	LG6	1.00±0.01	1.00±0.01
NZmsEB137749	LG7	1.01±0.01	1.01±0.02
TsuENH034	LG8	0.96±0.01	1.08±0.01
TsuENH097	LG9	1.02±0.02	0.91±0.04
Hi05b02	LG10	0.95±0.03	0.93±0.05
IPPN14	LG11	0.99±0.02	0.95±0.01
MEST011	LG12	0.99±0.02	0.99±0.04
CH02e02	LG13	0.95±0.03	0.89±0.02
TsuENH093	LG14	0.97±0.01	0.95±0.02
TsuENH007	LG15	0.95±0.00	0.98±0.02
Hi22f06	LG16	0.97±0.02	1.04±0.01
TsuENH002	LG17	0.95±0.02	1.02±0.08

The SSR-qPCR results are expressed as means ± standard deviations

Table S2 ΔRn values for the 17 pairs of SSR primers in 9 hybrid offspring of Q24 × 'Huabai No. 1'

SSR name	LG in loquat	Q24-1	Q24-2	Q24-3	Q24-4
TsuENH094	LG1	1.16±0.01	1.29*±0.04	1.38*±0.09	0.90±0.04
MEST028	LG2	1.03±0.03	1.10±0.02	1.05±0.04	1.49*±0.13
CH03g12	LG3	1.03±0.02	1.04±0.03	0.99±0.03	1.02±0.03
TsuENH044	LG4	1.02±0.08	1.15±0.03	1.00±0.10	0.85±0.10
NZmsCN898349	LG5	1.00±0.07	0.98±0.04	1.02±0.02	1.04±0.01
NZmsCO754252	LG6	1.00±0.01	1.00±0.01	1.00±0.04	1.00±0.03
NZmsEB137749	LG7	1.03±0.03	1.06±0.03	1.41*±0.01	1.01±0.00
TsuENH034	LG8	1.05±0.01	1.11±0.04	1.08±0.02	0.98±0.04
TsuENH097	LG9	1.00±0.01	0.99±0.04	1.04±0.02	1.55*±0.02
Hi05b02	LG10	0.98±0.06	1.04±0.04	1.06±0.03	1.06±0.05
IPPN14	LG11	0.96±0.01	0.99±0.01	1.03±0.00	1.02±0.00
MEST011	LG12	1.05±0.06	1.08±0.05	0.99±0.03	1.08±0.04
CH02e02	LG13	1.01±0.04	1.09±0.03	1.04±0.03	1.02±0.05
TsuENH093	LG14	0.97±0.04	0.97±0.02	1.02±0.02	1.03±0.03
TsuENH007	LG15	1.02±0.03	1.03±0.05	1.00±0.03	1.42*±0.01
Hi22f06	LG16	1.55*±0.04	1.08±0.02	1.02±0.01	0.85±0.03
TsuENH002	LG17	1.14±0.00	1.41*±0.05	0.86±0.02	0.74±0.08

The SSR-qPCR results are expressed as means ± standard deviations, * indicates LGs with abnormalities of chromosomes dosage, the SSR-qPCR results for Q24-1, Q24-2, Q24-3, Q24-4, Q24-5, Q24-7, Q24-8, Q24-9 correspond to the chromosome productions in Fig. 2

Table S2 (Continued)

SSR name	LG in loquat	Q24-5	Q24-6	Q24-7	Q24-8	Q24-9
TsuENH094	LG1	0.92±0.03	1.24±0.05	0.91±0.01	1.06±0.02	1.25*±0.02
MEST028	LG2	1.45*±0.08	1.56±0.04	1.06±0.07	1.46*±0.09	1.07±0.06
CH03g12	LG3	0.97±0.04	0.98±0.08	1.03±0.04	1.42*±0.07	1.01±0.06
TsuENH044	LG4	0.99±0.08	1.01±0.07	0.87±0.12	0.72±0.00	1.45*±0.05
NZmsCN898349	LG5	1.00±0.02	0.96±0.03	1.01±0.04	1.39*±0.09	0.99±0.03
NZmsCO754252	LG6	1.33*±0.02	1.00±0.04	1.00±0.02	1.00±0.02	1.00±0.03
NZmsEB137749	LG7	0.93±0.00	1.02±0.03	0.97±0.05	1.35*±0.05	1.41*±0.04
TsuENH034	LG8	1.51*±0.03	1.50±0.03	0.95±0.02	0.75±0.02	1.07±0.03
TsuENH097	LG9	0.94±0.02	1.00±0.07	1.04±0.03	1.55*±0.10	0.95±0.03
Hi05b02	LG10	1.42*±0.05	1.07±0.07	1.54*±0.07	1.49*±0.02	1.43*±0.03
IPPN14	LG11	0.98±0.02	1.41±0.04	0.96±0.01	0.91±0.05	1.44*±0.02
MEST011	LG12	0.97±0.03	1.31±0.04	1.25*±0.06	1.16±0.03	1.25*±0.06
CH02e02	LG13	0.98±0.02	1.08±0.04	1.52*±0.05	1.50*±0.13	1.45*±0.07
TsuENH093	LG14	0.99±0.03	0.97±0.02	1.49*±0.01	1.53*±0.03	1.46*±0.02
TsuENH007	LG15	1.01±0.01	1.03±0.03	1.47*±0.05	0.93±0.03	1.02±0.03
Hi22f06	LG16	0.98±0.03	1.03±0.02	1.30*±0.03	0.78±0.01	0.94±0.04
TsuENH002	LG17	1.00±0.01	1.23±0.07	0.70±0.00	0.76±0.01	1.30*±0.02

The SSR-qPCR results are expressed as means ± standard deviations, * indicates LGs with abnormalities of chromosomes dosage, the SSR-qPCR results for Q24-1, Q24-2, Q24-3, Q24-4, Q24-5, Q24-7, Q24-8, Q24-9 correspond to the chromosome productions in Fig. 2

Table S3 ΔRn values for the 17 pairs of SSR primers in 16 open-pollination progeny of triploid loquat strains (A313 and A322)

SSR name	LG in loquat	A313-1	A313-2	A313-3	A313-4
TsuENH094	LG1	1.08±0.06	1.46*±0.01	1.69±0.02	0.74±0.01
MEST028	LG2	1.15±0.09	1.04±0.06	1.71±0.08	0.79±0.05
CH03g12	LG3	0.93±0.13	1.03±0.02	1.01±0.03	0.70±0.03
TsuENH044	LG4	0.96±0.10	0.91±0.15	1.02±0.07	1.12±0.06
NZmsCN898349	LG5	1.01±0.04	0.93±0.08	1.00±0.03	1.07±0.03
NZmsCO754252	LG6	1.00±0.03	1.00±0.02	1.00±0.03	1.00±0.01
NZmsEB137749	LG7	1.00±0.01	1.44*±0.02	1.37±0.02	0.69±0.02
TsuENH034	LG8	0.93±0.02	0.95±0.02	0.82±0.05	0.32±0.01
TsuENH097	LG9	0.96±0.03	1.00±0.02	1.06±0.09	0.77±0.05
Hi05b02	LG10	1.13±0.05	0.76±0.04	1.30±0.07	0.62±0.01
IPPN14	LG11	1.40±0.06	0.96±0.02	0.97±0.04	0.67±0.06
MEST011	LG12	1.28±0.09	1.06±0.04	1.36±0.06	0.83±0.03
CH02e02	LG13	1.12±0.05	0.94±0.03	1.28±0.03	0.79±0.02
TsuENH093	LG14	1.05±0.05	1.44*±0.02	0.98±0.17	0.80±0.02
TsuENH007	LG15	0.92±0.01	0.95±0.04	0.85±0.08	0.63±0.00
Hi22f06	LG16	0.97±0.01	0.86±0.02	0.72±0.06	0.71±0.02
TsuENH002	LG17	1.22±0.22	0.90±0.01	1.16±0.12	0.79±0.08

The SSR-qPCR results are expressed as means ± standard deviations, * indicates LGs with abnormalities of chromosomes dosage, the SSR-qPCR results for A313-2, A313-5, A313-6, A322-1, A322-3, A322-4, A322-6, A322-7, A322-8, A322-9 correspond to the chromosome productions in Fig. 3

Table S3 (Continued)

SSR name	LG in loquat	A313-5	A313-6	A313-7	A322-1
TsuENH094	LG1	1.57*±0.02	1.47*±0.03	1.00±0.00	1.03±0.03
MEST028	LG2	1.10±0.01	0.98±0.03	1.08±0.04	1.03±0.07
CH03g12	LG3	0.99±0.05	0.95±0.04	0.97±0.04	0.99±0.04
TsuENH044	LG4	0.98±0.10	1.35*±0.08	0.85±0.13	1.03±0.10
NZmsCN898349	LG5	0.93±0.10	1.00±0.04	1.42±0.05	0.94±0.10
NZmsCO754252	LG6	1.00±0.03	1.00±0.01	1.00±0.00	1.00±0.02
NZmsEB137749	LG7	1.40*±0.04	1.30*±0.02	0.84±0.03	0.98±0.01
TsuENH034	LG8	0.91±0.02	0.93±0.07	0.38±0.03	1.05±0.03
TsuENH097	LG9	1.47*±0.03	0.89±0.04	1.67±0.02	0.90±0.05
Hi05b02	LG10	1.40*±0.02	0.96±0.07	1.42±0.05	0.96±0.05
IPPN14	LG11	0.93±0.01	1.38*±0.02	0.91±0.02	1.00±0.02
MEST011	LG12	1.29*±0.07	1.49*±0.06	1.04±0.04	1.02±0.05
CH02e02	LG13	1.11±0.06	0.88±0.02	1.36±0.04	0.91±0.03
TsuENH093	LG14	1.08±0.02	1.36*±0.02	1.13±0.03	0.99±0.03
TsuENH007	LG15	0.91±0.02	0.92±0.02	1.34±0.01	0.98±0.02
Hi22f06	LG16	0.89±0.08	0.97±0.04	0.98±0.03	0.94±0.01
TsuENH002	LG17	0.99±0.22	1.00±0.17	0.96±0.05	1.05±0.02

The SSR-qPCR results are expressed as means ± standard deviations, * indicates LGs with abnormalities of chromosomes dosage, the SSR-qPCR results for A313-2, A313-5, A313-6, A322-1, A322-3, A322-4, A322-6, A322-7, A322-8, A322-9 correspond to the chromosome productions in Fig. 3

Table S3 (Continued)

SSR name	LG in loquat	A322-2	A322-3	A322-4	A322-5
TsuENH094	LG1	1.12±0.05	0.99±0.03	0.95±0.04	0.68±0.05
MEST028	LG2	1.18±0.05	0.97±0.02	0.96±0.02	0.96±0.07
CH03g12	LG3	0.99±0.05	0.98±0.04	0.99±0.02	0.99±0.03
TsuENH044	LG4	1.09±0.09	0.98±0.06	0.93±0.14	0.49±0.02
NZmsCN898349	LG5	1.03±0.04	1.01±0.03	1.00±0.08	1.00±0.03
NZmsCO754252	LG6	1.00±0.02	1.00±0.02	1.00±0.01	1.00±0.04
NZmsEB137749	LG7	0.87±0.02	1.00±0.01	0.97±0.02	0.80±0.02
TsuENH034	LG8	0.89±0.03	0.93±0.07	0.91±0.06	0.56±0.04
TsuENH097	LG9	1.03±0.01	0.94±0.04	0.94±0.06	1.04±0.02
Hi05b02	LG10	1.11±0.05	0.99±0.04	0.93±0.01	1.13±0.05
IPPN14	LG11	0.96±0.01	1.06±0.01	0.94±0.02	1.00±0.02
MEST011	LG12	1.02±0.04	1.06±0.05	1.15*±0.06	0.93±0.04
CH02e02	LG13	1.13±0.05	0.94±0.03	0.90±0.01	0.88±0.05
TsuENH093	LG14	1.06±0.01	0.97±0.03	0.91±0.01	1.18±0.06
TsuENH007	LG15	0.89±0.03	1.00±0.02	0.90±0.02	0.82±0.01
Hi22f06	LG16	0.84±0.06	0.99±0.00	0.91±0.02	0.56±0.03
TsuENH002	LG17	1.19±0.03	1.11±0.02	0.84±0.03	0.47±0.02

The SSR-qPCR results are expressed as means ± standard deviations, * indicates LGs with abnormalities of chromosomes dosage, the SSR-qPCR results for A313-2, A313-5, A313-6, A322-1, A322-3, A322-4, A322-6, A322-7, A322-8, A322-9 correspond to the

chromosome productions in Fig. 3

Table S3 (Continued)

SSR name	LG in loquat	A322-6	A322-7	A322-8	A322-9
TsuENH094	LG1	1.03±0.03	0.98±0.04	1.18*±0.01	1.19*±0.01
MEST028	LG2	1.11±0.06	1.01±0.05	0.97±0.03	1.04±0.02
CH03g12	LG3	1.01±0.04	0.96±0.02	0.99±0.02	1.19*±0.04
TsuENH044	LG4	1.29*±0.08	0.91±0.00	0.91±0.18	0.95±0.09
NZmsCN898349	LG5	0.99±0.03	0.95±0.05	1.25*±0.05	1.23*±0.06
NZmsCO754252	LG6	1.00±0.01	1.00±0.03	1.00±0.01	1.00±0.05
NZmsEB137749	LG7	0.98±0.00	0.94±0.04	1.21*±0.03	1.08±0.01
TsuENH034	LG8	1.15*±0.02	1.17*±0.03	0.94±0.04	0.86±0.02
TsuENH097	LG9	1.02±0.02	1.16*±0.01	0.99±0.00	0.98±0.04
Hi05b02	LG10	1.02±0.04	0.98±0.05	0.93±0.07	1.07±0.01
IPPN14	LG11	0.95±0.01	0.97±0.03	1.03±0.01	1.03±0.02
MEST011	LG12	0.87±0.05	1.03±0.05	1.05±0.03	1.19*±0.02
CH02e02	LG13	1.01±0.03	0.95±0.03	1.18*±0.03	1.03±0.01
TsuENH093	LG14	1.02±0.02	0.99±0.03	1.17*±0.01	1.27*±0.04
TsuENH007	LG15	0.96±0.03	0.91±0.01	0.98±0.02	0.96±0.01
Hi22f06	LG16	1.11±0.02	0.84±0.11	0.93±0.04	1.09±0.00
TsuENH002	LG17	1.02±0.07	1.11±0.02	1.03±0.02	1.21*±0.02

The SSR-qPCR results are expressed as means ± standard deviations, * indicates LGs with abnormalities of chromosomes dosage, the SSR-qPCR results for A313-2, A313-5, A313-6, A322-1, A322-3, A322-4, A322-6, A322-7, A322-8, A322-9 correspond to the chromosome productions in Fig. 3
